# Validity and reliability of the executive function scale in Cuban university student

**DOI:** 10.3389/fpsyg.2025.1484883

**Published:** 2025-02-10

**Authors:** Diego D. Díaz-Guerra, Marena De La C. Hernández-Lugo, Carlos Ramos-Galarza, Yunier Broche-Pérez

**Affiliations:** ^1^Department of Psychology, Universidad Central “Marta Abreu” de Las Villas, Villa Clara, Cuba; ^2^Facultad de Psicología, Centro de Investigación MIST, Universidad Indoamérica, Quito, Ecuador; ^3^Prisma Behavioral Center, West Palm Beach, FL, United States

**Keywords:** executive functions, Cuban university students, UEF-1, validity, reliability

## Abstract

**Introduction:**

Executive functions are higher cognitive skills involved in planning, organization, decision-making, impulse control, and working memory. It is essential to have tools that allow for the accurate and reliable assessment of this construct in university students. This study aims to evaluate the validity and reliability of the Executive Functions Scale for University Students (UEF-1) in the Cuban population.

**Methods:**

A cross-sectional study was conducted in which an online survey was administered to 1,092 Cuban university students representing 14 of the country’s 16 provinces. Descriptive analyses, confirmatory factor analyses, and Pearson correlation analyses were used to assess the psychometric properties of the scale.

**Results:**

Significant correlations were obtained between the scale factors, and the original seven-factor structure was confirmed. The scale demonstrated good internal consistency and overall reliability (*α* = 0.91, *ω* = 0.91).

**Conclusion:**

The study provided evidence that the UEF-1 is a reliable and valid tool for assessing executive functions in Cuban university students. This measure provides a comprehensive understanding of the cognitive abilities and functioning of Cuban university students, allowing for the identification of specific areas of executive functioning that may benefit from additional support or intervention.

## 1 Introduction

Executive functions (EF) refer to a set of high-level cognitive processes that allow individuals to manage and regulate their thoughts, emotions, and behaviors in a goal-directed manner (Diamond, 2013). These processes are crucial for adaptive functioning across various life domains, including academic achievement, social interactions, and behavior regulation (Jacobson et al., 2011). EF encompasses several key cognitive components, including inhibition (the ability to suppress impulses), cognitive flexibility (the ability to shift attention and adapt to changing demands), planning and organization (the ability to formulate and execute strategies), and working memory (the ability to hold and manipulate information in the mind) (Karbach and Unger, 2014). These cognitive abilities play a critical role in academic success and behavior, with research indicating that strong executive functioning skills are positively associated with better school performance and fewer behavioral problems in children and adolescents (Jacobson et al., 2011).

The development of executive functions (EF) is a dynamic and ongoing process that evolves across childhood and adolescence, driven by both biological maturation and environmental influences (Best and Miller, 2010). Central to this development is the maturation of key brain regions, particularly the prefrontal cortex, which governs many EF processes, including planning, decision-making, inhibition, and cognitive flexibility (Laureys et al., 2021). The prefrontal cortex undergoes significant structural and functional changes during adolescence, which coincide with the refinement of EF abilities. This period is marked by increased synaptic pruning and the strengthening of neural connections, allowing for more efficient cognitive control and higher-order thinking (Larsen and Luna, 2018). As a result, adolescence is considered a critical window for the development and refinement of EF (Friedman and Robbins, 2022), during which the brain becomes better equipped to handle complex cognitive tasks and adapt to new demands.

However, this period of rapid cognitive development also makes adolescents particularly vulnerable to deficits in EF. Impairments in executive functioning during this stage can have profound consequences, contributing to difficulties in academic performance, poor impulse control, and challenges in emotional regulation and social interactions (Bailey et al., 2018). These deficits may manifest as problems with time management, task prioritization, and organization, making it harder for adolescents to navigate the academic and social pressures of school (Knouse et al., 2014). Furthermore, EF deficits during this critical developmental phase can lead to long-term difficulties in adulthood (Miller et al., 2012), as poor executive functioning in adolescence has been linked to higher rates of academic underachievement, mental health issues, and behavioral problems (Ferguson et al., 2021). Therefore, adolescence represents a key period not only for the natural development of EF but also for targeted interventions aimed at supporting these cognitive skills, which are crucial for academic success, social adaptation, and overall well-being (Tervo-Clemmens et al., 2023; Poon, 2018).

Given the importance of EF in both academic and behavioral outcomes, accurately assessing these functions is essential. Several tools have been developed to measure EF in various contexts. In this scenario, two well-known scales are the Behavior Rating Inventory of Executive Function (BRIEF2) (Gioia et al., 2015) and Barkley Deficits in Executive Functioning Scale (BDEFS) (Barkley, 2011).

The Behavior Rating Inventory of Executive Function, Second Edition (BRIEF2), is a widely used tool for assessing executive functioning across various settings (Gioia et al., 2015). It comprehensively measures everyday executive functioning difficulties by gathering ratings from parents, teachers, and other informants. The BRIEF2 covers a wide range of EF domains, including inhibition, shift, emotional control, initiation, working memory, and organization, offering valuable insights into how EF deficits manifest in real-world contexts. Its strength lies in its ability to capture EF impairments as they appear in natural environments, making it particularly useful for understanding how executive functioning affects day-to-day functioning and academic performance.

On the other hand, the Barkley Deficits in Executive Functioning Scale (Barkley, 2011), another prominent assessment tool, focuses on evaluating EF deficits within both home and school settings. It covers core areas such as self-regulation, organization, planning, and time management. One of the main advantages of the BDEFS is its emphasis on the impact of EF deficits on daily functioning, with a specific focus on how these deficits affect academic and social behaviors. It is particularly valuable for identifying issues related to time management and goal-directed behavior, which are common challenges in both educational and occupational contexts.

While the BRIEF2 and BDEFS provide valuable insights into executive functioning, their development within English-speaking contexts highlights the need for tools specifically designed for Spanish-speaking populations. The challenge lies in creating a scale that accurately evaluates executive functions within the unique cultural and educational frameworks of Spanish-speaking countries, particularly for university students in Latin America. This is crucial, as existing tools may not fully capture the nuances of the executive function challenges faced by these students. The University Executive Function Scale (UEF-1) represents a promising advancement in this area, having been created and validated specifically for Spanish-speaking university students (Ramos-Galarza et al., 2023). Its initial validation in Ecuadorian and Chilean populations demonstrated strong psychometric properties, including high reliability and validity (Tan et al., 2021). Developing such culturally sensitive tools is essential for accurately assessing and supporting the executive functioning of university students in Latin America.

However, the UEF-1 has not yet been evaluated in other Spanish-speaking contexts, particularly within the Cuban university population, where no specific EF assessment tools currently exist. In Cuba, generic tools designed for different clinical purposes have been adapted for evaluating EF, but these instruments may not fully capture the unique demands and challenges faced by university students in this context (Jiménez-Puig et al., 2019). Among the main challenges faced by university students in Latin American contexts are those related to language policy, political activism, neoliberal influences, educational inequities, mental health issues, and the need for pedagogical and technological adaptation (Cruz-Amarán et al., 2020; Hamel et al., 2016; Jhones and Larramendi, 2019; Mendoza, 2020; Ordorika, 2021). Furthermore, the tools used to assess executive functions in Cuba to date (e.g., Stroop, Tower of Hanoi, Bivalent Shape Test, Iowa Gambling Task) (Jiménez-Puig et al., 2019), according to Barkley’s extended phenotype model of executive functions (Barkley, 2012), focus on the instrumental-self-directed level and only evaluate the cross-temporal regulation of behavior during exceptionally brief assessment periods, without taking into account the self-regulation of the emotional and motivational components of executive functions.

This study aims to evaluate the psychometric properties of the University Executive Function Scale (UEF-1) in the Cuban university population. By assessing its reliability and validity, this research will contribute to a better understanding of the EF skills of Cuban students and provide a basis for developing targeted interventions to enhance these critical functions. Such interventions could play a significant role in supporting students’ academic success and emotional well-being.

## 2 Materials and methods

### 2.1 Study design and participants

This research was based on a cross-sectional study. An online survey was conducted using Google Forms^®^ from January to March 2024, which was distributed through WhatsApp groups, Facebook, email lists, and websites. No incentives were offered for participation. All Cuban citizens over 18 years’ old who were pursuing university studies within the country were eligible to participate. Regarding the inclusion criteria, it was established that all participants had to be active university students. Therefore, individuals with neurodevelopmental disorders, neurological disorders, or psychopathological conditions were excluded, thus ensuring that the sample consisted of students without conditions that could affect the study’s results.

The sample comprised 1,092 university students, with 39.6% identifying as male and 60.4% as female. Participants were aged between 18 and 25 years. A significant majority of the respondents were from the central region of the country, accounting for 93% of the total sample, with a notable concentration in the province of Villa Clara, which represented 76.8% of the participants. The remaining participants were from the occidental region (6.4%) and the oriental region of the country (0.7%) (see Table 1). The inclusion of an adequate number of participants in this research ensures a sufficient statistical power. The scale comprises 31 items, resulting in an average of 35.35 individuals per item.

**Table 1:** Participant demographic data (*n* = 1,092).

Demographic variable	Mean ± SD or n (%)
Age	20.05 ± 1.72
Gender	
Female	432 (39.6)
Male	660 (60.4)
Province	
Occidental Region	69 (6.4)
Pinar del Río	1 (0.1)
Artemisa	4 (0.4)
La Habana	61 (5.6)
Isla de la Juventud	1 (0.1)
Mayabeque	1 (0.1)
Matanzas	1 (0.1)
Central Region	1,016 (93.0)
Cienfuegos	44 (4.0)
Villa Clara	839 (76.8)
Sancti Spíritus	38 (3.5)
Ciego de Ávila	23 (2.1)
Camagüey	72 (6.6)
Oriental Region	7 (0.7)
Las Tunas	4 (0.4)
Granma	2 (0.2)
Santiago de Cuba	1 (0.1)

SE, standard deviation.

### 2.2 Measures

#### 2.2.1 Demographic Information

Demographic variables included age, gender, and province where university studies were being pursued.

#### 2.2.2 University Executive Function Scale

This scale was designed and validated for Spanish-speaking participants by Ramos-Galarza et al. (2023) in a sample of 1,373 Chilean and Ecuadorian students. It consists of 31 items and measures 7 executive functions. The subscales it assesses are: Conscious Monitoring of Responsibilities (F1 items 2, 8, 9, 15, and 27), Supervisory Attention System (F2 items 10, 14, 22, 28, and 13), Conscious Regulation of Behavior (F3 items 3, 11, 16, 17, 18, and 19); Behavior Verification for Learning (F4 items 20, 23, 24, and 30); Decision Making (F5 items 5, 12, and 21), Conscious Regulation of Emotions (F6 items 4, 25, 29, and 31), and Task-solving Element Management (F7 items 1, 6, 7, and 26). Responses are provided on a 5-point Likert scale (1 = strongly disagree to 5 = strongly agree) where high scores indicate greater executive functioning. The scale was specifically designed for administration in university populations, allowing for its application in individuals aged 18–35 years. Adequate internal consistency were found in the original version between *α* = 0.71 and 0.85. The seven executive functions proposed on the original scale correlated between *r* = 0.42 and 0.62. In the confirmatory factor analysis, good fit indices were obtained by these authors in the model of the seven executive functions χ^2^(413) = 1649.14, *p* = <0.001, CFI = 0.91, SRMR = 0.04 and RMSEA = 0.04.

The original version of the scale is aimed at Spanish-speaking students, so it was not necessary to translate the items. However, following the recommendations of Fenn et al. (2020) for the adaptation and translation of questionnaires, the original version of the technique was examined by three linguistics specialists affiliated with the Department of Linguistics at the Universidad Central “Marta Abreu” de Las Villas. The experts employed a comprehensive set of criteria to evaluate the linguistic suitability of the scale. These criteria included: linguistic accuracy (whether the language used in the scale accurately reflected the intended meanings of the items), cultural relevance (whether the language and examples used in the scale were culturally appropriate and relevant to the Cuban context), clarity of expression (whether the questions were easily understandable for students), and grammatical structure (grammatical correctness of the items). After a thorough review based on these criteria, the three experts concluded that the linguistic and grammatical structure of the scale was suitable for the Cuban context, and therefore, no modifications were necessary.

Additionally, a pilot study was conducted involving a sample of 50 students. This study aimed to assess the clarity, relevance, and overall suitability of the questions in relation to the language used within the Cuban context. Participants were invited to complete the scale and then provide feedback through a structured questionnaire that included open-ended questions. 98% of participants reported that they clearly understood the items, indicating that the language used was accessible and straightforward. Only 2% of participants identified specific items as confusing, primarily due to the use of certain technical terms that were unfamiliar to them. 96% of participants felt that the questions were relevant to their experiences and contexts, reaffirming the cultural appropriateness of the scale.

The findings of the pilot study confirmed the recommendations put forth by the linguistic experts. The majority of participants reported that the language was clear and easy to understand, with no significant issues identified. Furthermore, the feedback indicated that the questions were relevant to their experiences and contexts, reinforcing the cultural appropriateness of the scale. These results support the use of the questionnaire in the Cuban population without the need for additional linguistic or grammatical modifications.

### 2.3 Procedure and data analysis

Informed consent was obtained from all participants included in the study. All participants were fully informed that the process was conducted anonymously, and at no point were they required to provide any personal information. The ethics committee of the Department of Psychology of the Universidad Central “Marta Abreu” de Las Villas approved the study protocol (AN2JAN232024). All procedures performed in this study were following the ethical standards of the 1964 Helsinki Declaration. The scientific council of the Department of Psychology (Universidad Central Marta Abreu de Las Villas) approved the study under agreement number 2 on January 23, 2024.

The data were processed using the statistical software JASP version 0.18.3.0. Descriptive analyses were conducted to understand the characteristics of the participants. Additionally, a confirmatory factor analysis (CFA) was performed to evaluate the original factorial structure of the UEF-1, which encompasses seven dimensions of executive functions in the university context (Ramos-Galarza et al., 2023). Pearson correlation analyses were conducted to assess the relationship between the executive functions comprising the instrument, with correlation indices of *r* ≤ 0.10 considered small, *r* = 0.20 considered moderate, and *r* ≥ 0.30 considered large (Funder and Ozer, 2019).

Acceptable values for the comparative fit index (CFI) and the Tucker-Lewis index (TLI) were assumed to be between 0.90 and 0.95, while acceptable values for the standardized root mean square residual (SRMR) are those less than 0.08 (Hu and Bentler, 1999). As for the root mean square error of approximation (RMSEA), values below 0.08 were considered acceptable (Byrne, 2011; Schreiber et al., 2006). Cronbach’s *α* and McDonald’s *ω* were used to evaluate reliability, with values equal to or greater than 0.70 indicating good internal consistency (Dunn et al., 2013; Kelley, 2018; Shrestha, 2021).

To assess normality, the Kolmogorov–Smirnov test was employed. The *p*-values obtained were less than 0.05, indicating that the items of the UEF-1 were not normally distributed. The analysis of skewness and kurtosis revealed values exceeding ±1, providing conclusive evidence that the data does not adhere to a normal distribution (Mishra et al., 2019). Additionally, the Mardia’s test was conducted to assess the multivariate normality of the data. The results indicated a skewness coefficient of 6.86 (*p* < 0.001), suggesting a strong right skew in the distribution of the data. Additionally, the kurtosis coefficient was 80.45 (*p* < 0.001), indicating the presence of very heavy tails and a pronounced peak, which suggests a high concentration of extreme values. These results lead to the conclusion that the data do not follow a multivariate normal distribution (Wulandari et al., 2021). Considering the lack of normality in the sample, the weighted least squares mean, and variance adjusted estimator (WLSMV) were used for CFA (Xia and Yang, 2019).

## 3 Results

Table 2 displays the values corresponding to the analysis of means, standard deviation, correlations between the total items, and their loadings. The items showed above-average approval rates and considerable variation. Specifically, the mean scores for the items ranged from 3.30 to 4.53, with an overall average of approximately 4.01, suggesting that participants tended to agree with the presented statements. The standard deviations varied significantly, with values ranging from 0.35 to 1.90, reflecting a considerable degree of variability in the responses. Additionally, the loadings for the items varied from 0.38 to 0.84, with the majority of items exhibiting strong loadings above 0.60, all loadings were significant (*p* < 0.001). The item-test correlations values (r) for the items ranged from 0.35 to 0.63, demonstrating moderate to strong relationships among the items.

**Table 2:** Descriptive statistics, item-test correlations (r), and loadings of the UEF-1 items.

Items	*M*	*SD*	*r*	Loadings
F1 Conscious monitoring of responsibilities				
UEF2 Puedo terminar una tarea universitaria cuando es muy larga/*I can finish a university assignment when it is very long.*	4.01	1.04	0.50	0.70
UEF8 Puedo realizar las tareas universitarias de forma independiente y sin ayuda de los demás/*I can complete college assignments independently and without help from others.*	3.80	1.90	0.43	0.63
UEF9 Logro realizar exitosamente mis trabajos de la universidad/*I successfully complete my university assignments*.	4.17	0.89	0.55	0.79
UEF15 Puedo realizar mis trabajos sin que alguien me supervise/*I can do my assignments without someone supervising me.*	4.14	0.99	0.41	0.61
UEF27 Termino mis tareas universitarias a tiempo/*I finish my college assignments on time.*	4.07	1.03	0.54	0.77
F2 Supervisory Attention System				
UEF10 Tengo buena concentración/*I can concentrate well.*	3.60	1.21	0.60	0.77
UEF14 Soy capaz de mantener la atención en una actividad/*I can maintain my attention on an activity*.	3.96	1.07	0.59	0.74
UEF22 Me es fácil concentrarme en mis actividades universitarias/*It is easy for me to concentrate on my college activities.*	3.70	1.11	0.63	0.81
UEF28 Mantengo buenos hábitos de estudio/*I maintain good study habits.*	3.47	1.26	0.57	0.73
UEF13 Me concentro en mis actividades universitarias, dejando de lado las cosas irrelevantes/*I focus on my university activities, leaving irrelevant things aside.*	3.35	1.21	0.59	0.74
F3 Conscious regulation of behavior				
UEF3 Actúo siempre pensando y reflexionando las consecuencias de mis actos/*I always act thinking and reflecting on the consequences of my actions.*	3.87	1.17	0.46	0.56
UEF11 Puedo estar quieto/a y tranquilo/a mientras espero/*I can be still and calm while I wait.*	3.30	1.43	0.42	0.54
UEF16 Me es fácil comportarme adecuadamente en las reuniones sociales/*It is easy for me to behave appropriately in social gatherings.*	4.53	0.84	0.47	0.66
UEF17 Cuando alguien me lo pide, puedo dejar con facilidad de hacer algo que me distrae/*When someone asks me to, I can easily stop doing something that* distracts me.	4.01	1.07	0.47	0.59
UEF18 Dejo hablar a los demás, sin hacer interrupciones/*I let others speak, without interrupting.*	4.13	1.05	0.38	0.49
UEF19 Puedo anticipar las consecuencias de mis actos/*I can anticipate the consequences of my actions.*	3.95	1.04	0.35	0.46
F4 Verification of behavior to learn				
UEF20 Verifico que mis tareas universitarias estén bien realizadas y sin errores, antes de presentarlas al profesor/*I verify that my university assignments are well done and without errors, before giving them to the professor.*	4.12	1.08	0.52	0.81
UEF23 Reviso la ortografía y redacción de mis tareas universitarias antes de finalizarlas/*I check the spelling and wording of my college assignments before I finish them.*	4.17	1.23	0.39	0.67
UEF24 Recuerdo llevar a casa las tareas, materiales o papeles de la universidad/*I remember to take home assignments, materials, or college papers.*	4.06	1.22	0.46	0.71
UEF30 Al finalizar una actividad universitaria, verifico que haya logrado lo planificado/*At the end of a university activity, I verify that I have achieved what I planned.*	4.06	1.06	0.56	0.81
F5 Decision making				
UEF5 Tengo la capacidad para tomar decisiones en forma independiente/*I can make decisions independently.*	4.29	0.95	0.43	0.64
UEF12 Tengo la capacidad para resolver problemas en la universidad como en mi vida personal/*I can solve problems at university as well as in my personal life.*	4.12	0.96	0.55	0.80
UEF21 Puedo tomar decisiones sin dificultad, incluso ante las cosas más complicadas/*I can make decisions without difficulty, even in the most complicated things.*	3.85	1.07	0.51	0.73
F6 Conscious regulation of emotions				
UEF4 Regulo adecuadamente mis emociones/*I properly regulate my emotions.*	3.65	1.17	0.49	0.77
UEF25 Mantengo la calma con facilidad/*I can keep calm easily.*	3.65	1.20	0.45	0.69
UEF29 Tengo un estado de ánimo estable/*My moods are stable.*	3.66	1.29	0.53	0.82
UEF31 Soy capaz de regular mis emociones/*I can regulate my emotions.*	3.78	1.20	0.50	0.84
F7 Management of elements to solve tasks				
UEF1 Tengo facilidad para recoger y dejar ordenadas mis cosas cuando se me pide que lo haga/*It is easy to collect and leave my things organized when asked to do so.*	4.01	1.11	0.50	0.75
UEF6 Tengo mis cosas en el lugar adecuado y en orden/*I have my things in the right place and organized.*	3.65	1.31	0.51	0.78
UEF7 Tengo facilidad para encontrar rápidamente mis materiales al buscarlos en mi cuarto o escritorio/*I have an easy time finding my materials by looking for them in my room or desk.*	3.94	1.25	0.46	0.71
UEF26 Recojo mi desorden sin que otros lo hagan por mí/*I pick up my mess without others having to do it for me*	4.13	1.64	0.41	0.66

M, mean; SD, standard deviation; r, correlation index.

Furthermore, the analysis revealed statistically significant correlations of moderate to large magnitudes between the factors of the scale, as shown in Table 3. Specifically, the correlations between the factors ranged from 0.235 to 0.614, with all correlations being statistically significant at the *p* < 0.001 level.

**Table 3:** Correlation matrix between executive functions.

	F1	F2	F3	F4	F5	F6	F7
F1	–						
F2	0.614***	–					
F3	0.426***	0.546***	–				
F4	0.511***	0.559***	0.427***	–			
F5	0.473***	0.486***	0.475***	0.324***	–		
F6	0.300***	0.443***	0.558***	0.235***	0.508***	–	
F7	0.400***	0.435***	0.469***	0.446***	0.375***	0.357***	–

F1, Conscious monitoring of responsibilities; F2, Supervisory attention system; F3, Conscious regulation of behavior; F4, Verification of behavior to learn; F5, Decision making; F6, Conscious regulation of emotions; F7, Management of elements to solve tasks; ***p < 0.001.

The seven-factor solution demonstrated satisfactory fit indices, indicating a robust model that aligns well with the data. Specifically, the chi-square statistic was χ^2^(413) = 1823.68, with a significance level of *p* < 0.001, suggesting that the model fits the observed data significantly better than a null model. Additionally, the Comparative Fit Index (CFI) was reported at 0.981, and the Tucker-Lewis Index (TLI) was 0.979, both well above the commonly accepted threshold of 0.90, indicating a good fit. The Root Mean Square Error of Approximation (RMSEA) was calculated at 0.056, with a 90% confidence interval ranging from 0.053 to 0.059, and a significance level of *p* < 0.001. The value we obtained for the Standardized Root Mean Square Residual (SRMR) was 0.05, which is considered acceptable.

These indices collectively suggest that the model adequately captures the underlying structure of the data. Consequently, the initial solution of the seven proposed factors in the original scale is confirmed. Given these strong fit indices, it was deemed unnecessary to test a second-order model that would consider a central factor of executive functions. This decision aligns with the findings from the original validation of the scale, which indicated that such a model did not yield an acceptable fit.

Additionally, reliability estimates were high for the total score (*α* = 0.91, *ω* = 0.91). Regarding factor analysis, there were predominantly medium values in conscious monitoring of responsibilities (*α* = 0.76, *ω* = 0.77), supervisory attention system (*α* = 0.83, *ω* = 0.83), behavior verification for learning (*α* = 0.76, *ω* = 0.76), decision making (*α* = 0.70, *ω* = 0.70), conscious regulation of emotions (*α* = 0.82, *ω* = 0.82), and task-solving element management (*α* = 0.76, *ω* = 0.76). There were medium-low values for the conscious regulation of behavior dimension (*α* = 0.65, *ω* = 0.65).

We calculated normative scores for the executive function scale adapted to the Cuban university population (UEF-C; see Table 4) to create a benchmark for interpreting individual performance. To derive these normative data, we systematically classified the factor scores into five quartiles based on the percentile calculations. We established cut-off points both overall and by gender, which allows for a more nuanced understanding of how different groups perform on the scale. The following cut-off points and classifications were established:

- *Very below average:* pc < 5 (5th percentile)
- *Below average:* 5 ≤ pc < 25 (5th–25th percentile)
- *Average:* 25 ≤ pc < 75 (25th–75th percentile)
- *Above average*: 75 ≤ pc < 95 (75th–95th percentile)
- *Very above average:* pc ≥ 95 (95th percentile)

**Table 4:** UEF-C cutoff used to classify the factors scores.

Indicator	F1			F2			F3			F4			F5			F6			F7		
	G	M	F	G	M	F	G	M	F	G	M	F	G	M	F	G	M	F	G	M	F
M	20.2	19.66	20.58	18.1	17.5	18.4	23.8	23.5	23.9	16.4	15.3	17.1	12.2	12.5	12.1	14.7	15.4	14.2	15.7	14.9	16.2
SD	3.65	3.95	3.39	4.57	4.66	4.47	4.06	4.14	4.00	3.51	3.90	3.03	2.37	2.32	2.40	3.94	3.72	4.01	3.71	3.79	3.58
	Percentiles																				
5th	13	13	14	10	9	10	16	16	16	9	8	11	8	8	7	7	8	6	8	8	9
10th	15	14	16	12	11	12	19	18	19	11	10	13	9	10	9	9	10	8	10	10	11
15th	16	15	17	13	13	14	20	20	20	13	11	14	10	10	10	10	12	9	12	11	13
20th	17	16	18	14	14	15	21	20	21	14	12	15	10	11	10	11	12	11	13	12	14
25th	18	17	19	15	15	16	22	21	22	14	13	16	11	11	11	12	14	12	13	13	14
30th	19	18	19	16	15	17	22	22	22	15	14	16	11	12	11	13	14	13	14	13	15
35th	19	19	20	17	16	17	23	23	23	16	14	17	12	12	12	14	15	14	15	14	16
40th	20	19	20	18	17	18	23	23	24	16	15	17	12	12	12	15	15	14	16	14	16
45th	20	20	21	18	17	19	24	24	24	17	15	17	12	13	12	15	16	15	16	15	17
50th	21	20	21	19	18	19	24	24	24	17	16	18	13	13	12	16	16	15	17	15	17
55th	21	21	21	19	19	20	25	24	25	18	16	18	13	13	13	16	16	16	17	16	18
60th	22	21	22	20	19	20	25	25	25	18	17	19	13	14	13	16	17	16	18	16	18
65th	22	22	22	20	20	20	26	25	26	19	17	19	14	14	13	17	17	16	18	17	18
70th	23	22	23	21	20	21	26	26	26	19	18	20	14	14	14	17	18	17	18	18	19
75th	23	23	23	21	21	22	27	27	27	20	19	20	14	14	14	18	18	17	19	18	19
80th	24	23	24	22	22	22	27	27	27	20	19	20	14	15	14	18	19	18	19	19	19
85th	24	24	24	23	22	23	28	28	28	20	20	20	15	15	15	19	19	18	20	19	20
90th	25	25	25	24	24	24	29	28	29	20	20	20	15	15	15	20	20	19	20	20	20
95th	25	25	25	25	25	25	30	30	30	20	20	20	15	15	15	20	20	20	20	20	20
100th	25	25	25	25	25	25	30	30	30	20	20	20	15	15	15	20	20	20	20	20	20

SD, Standard Deviation; M, mean; G, General Punctuation; M, Male’s Punctuations; F, Female’s Punctuations; F1, Conscious monitoring of responsibilities; F2, Supervisory attention system; F3, Conscious regulation of behavior; F4, Verification of behavior to learn; F5, Decision making; F6, Conscious regulation of emotions; F7, Management of elements to solve task.

## 4 Discussion

The purpose of this research was to evaluate the psychometric properties of the UEF-1 in Cuban university students. The results obtained confirm the factorial structure of the original version composed of seven executive functions (Ramos-Galarza et al., 2023).

This research found an adequate fit of the seven-factor model originally proposed by Ramos-Galarza et al. (2023). The fit indices found by these authors (CFI = 0.91, SRMR = 0.04, and RMSEA = 0.04) resemble those obtained in the Cuban validation (CFI = 0.981; TLI = 0.979; RMSEA = 0.056,). These fit indices provide evidence of the validity of the model and the scale’s quality in terms of its structure and ability to measure the desired constructs (Finch, 2020; Barnes and Forde, 2021; Koran, 2020; McNeish and Wolf, 2023).

Similarly to the initial design of the scale, in this research, statistically significant and directly proportional correlations of large magnitudes were found between the evaluated executive functions (*r* = 0.23–0.61 in the Cuban population and *r* = 0.27–0.62 in the Ecuadorian and Chilean population) (Ramos-Galarza et al., 2023). The correlation analyses reveal that the factors are interrelated, suggesting that they may influence each other within the context of the studied construct (Schober et al., 2018; Tavakol and Wetzel, 2020).

The total reliability estimates of the original scale were similar to those obtained in the Cuban university population. However, the factor estimates in Cuban students showed slightly lower values than those obtained in the original scale (Ramos-Galarza et al., 2023). The main difference was observed in the dimension of conscious regulation of behavior (*α* = 0.65, *ω* = 0.65) compared to the original sample (*α* = 0.76 and *ω* = 0.74). However, all values are within the acceptable range of reliability (Dunn et al., 2013; Fu et al., 2022).

This research aligns with previous studies on executive functions in students. For example, Escolano-Pérez et al. (2022) and Ramos-Galarza et al. (2023) found that evaluating executive functions through behavioral observation is a reliable method for assessing these mental abilities. This approach allows for observation within a real-world context, providing insights into the functioning of executive functions such as inhibitory control, cognitive flexibility, working memory, emotional regulation, and decision-making. These converging results highlight the importance of incorporating executive function development into university programs. By providing students with both knowledge and practical strategies for utilizing these skills, universities can contribute to improved academic performance, daily functioning, and future job prospects, as suggested by Prosen and Vitulic (2014) and Kamradt et al. (2021).

An executive function assessment scale specifically designed for the university context offers several advantages over existing scales that evaluate executive functions or frontal lobe abilities in the general adult population. Examples of such general scales include the Adult Executive Functioning Inventory (ADEXI), the Scale Assessment of Executive Functions-Adult (SAEF-A), and the EOCL-1 Scale for Assessing Executive Functions (El Houari et al., 2024; López et al., 2022; Ramos-Galarza et al., 2021). Firstly, the UEF-1 scale focuses on assessing the capabilities of executive functions rather than the deficits in these mental abilities, which is the primary concern of scales developed for the adult population. Secondly, the UEF-1 scale evaluates real-life situations faced by university students, providing a context in which the practical application of executive functions can be observed. Lastly, its free accessibility allows researchers worldwide to enhance the instrument, and, as demonstrated in this study, its psychometric properties can be analyzed in new contexts.

The present study has relevant implications regarding the assessment of executive functions in real-life situations of Cuban university students. First, the availability of the UEF-C as a reliable and valid instrument for the Cuban context provides a valuable tool for assessing executive functions in real-life situations of Cuban university students. This instrument can facilitate the profiling of performance, inform the development of tailored neuropsychological interventions, and contribute to enhancing students’ executive function skills, ultimately improving their academic and daily functioning. Additionally, our study replicates the findings from the original study, a critical aspect of ensuring that (neuro)psychological measures are applicable and meaningful across different cultural contexts. In this sense, our results play a crucial role in advancing our knowledge of cross-cultural variations in psychological constructs and ensuring the validity of psychological measures across diverse populations.

Despite the contributions of this study, several limitations warrant consideration. We were unable to conduct convergent validity tests with other measures of executive functioning due to the requirement of paid usage licenses for the most commonly used tools in this area. The lack of financial resources in Cuba limits our access to paid licenses for questionnaires and other assessment instruments, which in turn restricts our ability to make comparisons with established measures. It is important to highlight that this limitation does not diminish the relevance of our research; rather, it underscores the need to develop and validate accessible tools for assessing executive functioning in contexts where resources are scarce. In this sense, the UEF-1 emerges as a valuable alternative, not only for its free availability to Spanish-speaking populations but also for its potential to be used in economically constrained environments. Additionally, the exclusive use of self-reported data, the reliance on predominantly student samples, and the use of snowball sampling for participant recruitment may limit the generalizability of our findings. Future research should aim to address these limitations by incorporating diverse samples, employing objective measures of executive functions, and exploring the nomological validity and temporal stability of the UEF-1 scores over time.

## 5 Conclusion

In conclusion, this study provides valuable insights into the psychometric properties of the UEF-1 in Cuban university students, confirming its validity and reliability as a measure of executive functions in this population. By advancing our understanding of executive functions and their assessment in diverse cultural contexts, this research contributes to the broader field of neuropsychological assessment and intervention, highlighting the importance of promoting executive function skills to support student’s academic success and daily functioning.

## Data Availability

The datasets presented in this study can be found in online repositories. The names of the repository/repositories and accession number(s) can be found in the article/supplementary material.
